# Burden of alcohol use disorders in China and the regions with different income levels over the world

**DOI:** 10.7189/jogh.11.08011

**Published:** 2021-12-30

**Authors:** Xiang Qu, Mi Liu, Changrong Ke, Juanjuan Liang, Yuanze Du, Liqun Yao, Juanjuan Li, Guixian Mu, Shiwei Liu, Chunping Wang

**Affiliations:** 1 School of Public Health, Weifang Medical University, Weifang, China; 2 Hospital infection management office, The Second People's Hospital of Lianyungang Lianyungang, China; 3 Tobacco Control office, Chinese Center for Disease Control and Prevention, Beijing, China

## Abstract

**Background:**

Alcohol use disorders (AUD) has long been one of the most disability mental disorders and a major cause of health loss.

**Methods:**

Based on open access data from the 2019 Global Burden of Disease (GBD 2019) study, we extracted data of years lived with disability (YLD), years of life lost (YLL) and disability-adjusted life years (DALY) to describe the changes of AUD burden over the period of 1990-2019 stratified by sex in globe, high-income countries (HICs), upper-middle income countries (UMCs), lower-middle income countries (LMCs), low-income countries (LICs) and China. We used Joinpoint regression model to fit the changing trend of years. And pairwise comparison was applied to test the coincidence parallelism and judge whether the difference of the trend among different regions is statistically significant.

**Results:**

LMCs experienced the largest changes in the YLD rate of AUD from 1990 to 2019 (average annual percent change (AAPC) = -0.7, 95% confidence interval (CI) = -0.8, -0.7, *P* < 0.05), with China experienced a higher increase in 1990 to 1993 (annual percent change (APC) = 3.8, 95% CI = 3.2, 4.4, *P* < 0.05) than other regions, and the rate of decline in China from 1996 to 2002 (APC = -3.4, 95% CI = -3.6, -3.1, *P* < 0.05) was higher than that in other regions. UMCs experienced the largest changes in the YLL rate of AUD from 1990 to 2019 (AAPC = -1.1, 95% CI = -1.6, -0.6, *P* < 0.05), with a larger decline in 2004 to 2012 (APC = -6.2, 95% CI = -6.7, -5.7, P < 0.05) than other regions, and China experienced a larger increase in the rate of YLL from 1999 to 2004 (APC = 9.2, 95% CI = 8.5, 9.9, P < 0.05) than other regions. LMCs experienced the largest changes in the DALY rate of AUD from 1990 to 2019 (AAPC = -0.9, 95% CI = -1.0, -0.8, P < 0.05), with a larger decline in 2006 to 2010 (APC = -3.3, 95% CI = -3.6, -2.9, P < 0.05) than other regions, and UMCs showed a larger increase in the rate of DALY from 1990 to 1994 (APC = 4.5, 95% CI = 3.8, 5.1, P < 0.05) than other regions.

**Conclusions:**

Given the large variations in AUD burden of disease by income level, future strategies to prevent and reduce the burden should be developed and implemented based on country-specific development status.

Alcohol use disorders (AUD) are diseases in which the drinker is unable to control the time or amount of alcohol consumed [1]. The repeated consumption of alcohol in large quantities over a long period of time causes damage to physical or mental health [2]. It has the characteristics of high prevalence rate, great harm, and heavy burden of disease [3,4]. Persistent heavy drinking not only damages the cardiovascular, gastrointestinal and immune systems, but also increases the risk of heart disease, stroke and liver cirrhosis [5,6]. According to the 2019 Global Burden of Disease (GBD 2019), the globe AUD disability-adjusted life years (DALY) in 2019 was about 1.7019 million people per year [7], and the report of the World Health Organization (WHO) points out, an estimated 4.9% of the world's adult population (240 million people) suffer from alcohol use disorder (7.8% of men and 1.5% of women), with alcohol causing an estimated 257 disability-adjusted life years lost per 100 000 population [8]. According to the Chinese epidemiological survey, the prevalence rate of AUD is 2.4% [9], which leads to a significant economic and social burden [10], and has become a pressing national public health issue [11]. AUD is treatable, and timely implementation of targeted interventions improves outcomes [12]. Alcohol use disorders are associated with socio-economic level and studies have shown that people of low socio-economic status are at higher risk of developing alcohol use disorders than those of high socio-economic status [13]. Considering the lack of comprehensive evaluation studies on the current situation and changing trend of AUD burden in the regions with different income levels over the world, our current study uses the latest research results of GBD 2019 to explore the AUD burden and its changing trend in China and the regions with different income levels over the world from 1990 to 2019 [7,14,15]. It provides a basis for China to determine AUD disease prevention and control strategies and the order of medical resources allocation in AUD diseases according to their severity.

## METHODS

### Data source

The GBD study used unified and standard methods to ensure that the results were comparable, and regionally and nationally representative [16]. We extracted AUD data from the 2019 GBD study from 1990 through 2019. All data obtained in the present study were publicly available at the Institute for Health Metrics and Evaluation (IHME) website and can be accessed with open online tools (http://www.healthdata.org/results/data-visualizations; http://ghdx.healthdata.org/gbd-resultstool). GBD is an ongoing global collaboration that uses all available epidemiological data to provide a comparative assessment of health loss from 369 diseases and injuries, and 87 risk factors across 204 countries and territories [7,14,15,17]. From GBD study 2019, we obtained data on years lived with disability (YLD), years of life lost (YLL), and disability-adjusted life years (DALY) and respective age-standardized rates of AUD from 1990 to 2019. The cause-of-death data used by GBD to estimate the burden of disease in China are mainly from China Disease Surveillance System, China Maternal and Child Health Surveillance Network, China Health Statistical Yearbook and Chinese Center for Disease Control and Prevention, etc. Data on incidence and prevalence are mainly derived from disease surveillance, national health service surveys and published literature studies [18]. GBD 2019 uses deidentified, aggregated data, and a waiver of informed consent was reviewed and approved by the University of Washington Institutional Review Board [19]. The study included data on the burden of AUD in China and the regions with different income levels over the world in 1990,2005 and 2019.

### Definition

#### AUD

Alcohol use disorders (AUD) is a problematic pattern of alcohol use leading to clinically significant impairment or distress [20]. AUD is often diagnosed clinically according to Diagnostic and Statistical Manual for Mental Disorders, 5th edition (DSM-5), and there are 11 diagnostic criteria in total. AUD requires that ≥2 diagnostic criteria to be met within a 12-month period, with mild AUD: 2-3 criteria; moderate AUD: 4-6 criteria; and severe AUD: 7-11 criteria [13,21]. AUD is classified as diagnosis codes F-10 by the International Statistical Classification of Diseases (Tenth Revision) (ICD-10) [22,23].

#### YLD

Years lived with disability (YLD) represents healthy life-years lost in survivors, were estimated by multiplying prevalence by disability weights, YLD = Prev × DW [24].

#### YLL

Years of life lost(YLL) were estimated by multiplying age-specific AUD deaths by a reference life expectancy, YLL = N × L [25].

#### DALY

Disability-adjusted life years (DALY) is the sum of two components, which are years of life lost to premature mortality and years of life lived with disability, DALY = YLD+YLL [26].

#### Uncertainty Interval

For YLD, YLL, and DALY, the corresponding 95% uncertainty intervals (UI) were calculated using the 2.5th and 97.5th estimates in posterior simulation of 1000 ordered draws, with the aim of examining uncertain distributions deriving from random and systematic errors [27]. Significant differences were defined as no overlap of their 95% UIs between any 2 estimates.

### Statistical analysis

To characterize the temporal trends across regions and countries, age-standardized rates (per 100 000) were computed using the globe age-standard population constructed by the WHO. The total rate of change from 1990 to 2019, average annual rate of change from 1990 to 2005 and from 2005 to 2019 were used to analyze the trend of AUD disease burden.


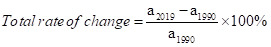
,



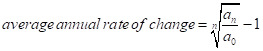



We used Joinpoint 4.9.0.0 software to do Joinpoint regression analysis [28], taking time as independent variable and the age-standardized rate of YLD, YLL and DALY as dependent variable respectively. And pairwise comparison was applied to test the coincidence parallelism and judge whether the difference of the trend among different regions is statistically significant [29]. The test level is α = 0.05.

### Geographical estimation

Based on income levels categorized by the World Bank, the countries and territories were stratified into four groups, which classified as high-income countries (HICs), upper-middle income countries (UMCs), lower-middle income countries (LMCs), and Low-income countries (LICs), respectively.

## RESULTS

### Sex-specific

In terms of gender, the age-standardized YLD rate, YLL rate, and DALY rate in China and the regions with different income levels over the world were all lower for females than for males (**Table 1**).

**Table1:** Burden of disease associated with alcohol use disorders in China and the regions with different income over the world from 1990 to 2019

Year	YLD (1/100 000)			YLL (1/100 000)			DALY (1/100 000)		
**Male**	**Female**	**Both**	**Male**	**Female**	**Both**	**Male**	**Female**	**Both**
**Globe**									
1990	243.7 (165.3,345.0)	67.9 (45.0,94.9)	155.9 (105.6,220.1)	166.5 (152.0,174.7)	33.9 (32.5,35.0)	100.3 (92.4,104.7)	410.2 (331.0,513.6)	101.7 (78.9,128.7)	256.2 (205.3,321.6)
2005	222.8 (151.1,315.2)	66.1 (43.9,92.1)	144.2 (97.4,203.7)	188.7 (172.6,196.5)	35.2 (34.4,36.8)	111.9 (104.2,116.2)	411.5 (337.7,506.5)	101.3 (79.0,127.3)	256.1 (208.1,315.8)
2019	205.1 (139.0,291.0)	58.9 (39.3,82.5)	131.8 (89.0,186.7)	130.9 (109.7,140.9)	20.9 (19.0,22.6)	75.6 (64.7,80.7)	336.0 (267.6,422.1)	79.8 (59.9,103.6)	207.3 (163.7,261.7)
Total rate of change (%)	-0.16	-0.13	-0.15	-0.21	-0.38	-0.25	-0.18	-0.22	-0.19
Average annual rate of change (%):									
1990-2005	-0.60	-0.18	-0.52	0.84	0.26	0.73	0.02	-0.03	0.00
2005-2019	-0.59	-0.82	-0.64	-2.58	-3.67	-2.76	-1.44	-1.69	-1.50
**HICs:**									
1990	295.8 (195.8,416.6)	119.6 (79.1,167.9)	207.2 (137.0,289.7)	152.8 (150.4,155.5)	32.9 (32.1,33.7)	91.6 (90.3,93.1)	448.6 (348.3,570.0)	152.5 (112.0,200.3)	298.9 (228.8,381.8)
2005	270.2 (180.9,376.1)	119.0 (79.2,163.8)	194.7 (130.6,269.4)	154.7 (152.4,157.5)	38.2 (37.4,39.1)	95.8 (94.6,97.3)	424.8 (334.8,530.4)	157.2 (117.5,202.2)	290.5 (225.8,365.0)
2019	256.6 (171.4,360.4)	118.4 (78.6,164.7)	188.6 (126.1,263.6)	140.6 (132.4,148.2)	39.5 (37.4,41.4)	90.1 (85.6,94.2)	397.2 (311.5,503.7)	157.9 (118.3,204.4)	278.7 (216.2,354.8)
Total rate of change (%)	-13.23	-0.98	-9.00	-8.00	20.12	-1.67	-11.45	3.57	-6.75
Average annual rate of change (%):									
1990-2005	-0.60	-0.03	-0.41	0.08	1.00	0.30	-0.36	0.20	-0.19
2005-2019	-0.37	-0.04	-0.23	-0.68	0.25	-0.44	-0.48	0.03	-0.30
**UMCs:**									
1990	253.9 (172.7,358.2)	66.0 (43.9,92.6)	160.2 (108.2,225.2)	195.0 (181.6,205.2)	46.1 (43.1,48.7)	121.2 (113.8,126.9)	448.9 (366.6,555.7)	112.1 (90.0,139.5)	281.4 (229.2,348.9)
2005	224.0 (151.6,316.3)	67.9 (45.0,95.0)	145.6 (98.1,204.8)	249.2 (234.4,263.5)	49.1 (47.6,52.1)	149.0 (142.6,157.3)	473.2 (398.5,568.7)	116.9 (93.5,144.5)	294.6 (245.8,355.5)
2019	225.4 (152.5,320.9)	61.6 (40.6,86.7)	143.3 (96.3,203.2)	148.5 (122.5,165.0)	23.5 (19.8,27.1)	85.6 (72.2,94.6)	373.8 (296.6,470.3)	85.1 (64.5,111.3)	228.9 (181.5,288.8)
Total rate of change (%)	-11.23	-6.64	-10.59	-23.87	-49.09	-29.34	-16.72	-24.09	-18.66
Average annual rate of change (%):									
1990-2005	-0.83	0.18	-0.64	1.65	0.42	1.39	0.35	0.28	0.31
2005-2019	0.04	-0.69	-0.12	-3.63	-5.13	-3.88	-1.67	-2.24	-1.79
**LMCs**									
1990	210.4 (142.1,299.5)	41.3 (27.4,58.2)	127.2 (85.9,181.1)	149.3 (114.7,166.2)	21.3 (20.3,22.4)	86.6 (68.9,95.3)	359.7 (285.5,448.6)	62.6 (48.7,79.7)	213.8 (169.6,265.9)
2005	207.1 (140.1,295.0)	42.5 (28.1,60.2)	125.2 (84.1,177.9)	147.6 (121.0,157.5)	18.5 (17.6,19.4)	83.6 (70.2,88.6)	354.8 (283.1,443.9)	61.0 (46.7,78.5)	208.8 (166.2,262.5)
2019	168.7 (115.6,238.3)	36.8 (24.6,52.0)	102.5 (69.9,145.3)	114.6 (92.0,132.1)	10.1 (8.8,11.6)	62.1 (50.7,71.1)	283.3 (226.0,356.4)	46.9 (35.0,62.5)	164.6 (130.3,208.7)
Total rate of change (%)	-19.80	-10.90	-19.38	-23.22	-52.56	-28.26	-21.22	-25.08	-22.97
Average annual rate of change (%)									
1990-2005	-0.10	0.20	-0.10	-0.07	-0.95	-0.23	-0.09	-0.17	-0.16
2005-2019	-1.45	-1.03	-1.42	-1.79	-4.21	-2.10	-1.59	-1.86	-1.68
**LICs:**									
1990	195.7 (130.8,279.1)	56.2 (37.4,79.9)	123.1 (82.5,175.0)	88.4 (65.0,107.1)	19.3 (16.4,22.3)	52.8 (41.1,62.6)	284.1 (212.2,370.4)	75.5 (56.2,99.1)	175.9 (132.5,229.3)
2005	185.6 (125.4,262.7)	54.9 (36.7,79.4)	118.3 (79.9,168.0)	79.4 (63.4,92.1)	16.5 (14.4,18.7)	47.0 (39.3,53.8)	265.0 (203.2,344.7)	71.5 (52.5,95.8)	165.3 (125.3,216.9)
2019	199.6 (133.9,282.6)	55.0 (36.4,78.8)	125.0 (84.1,177.3)	73.4 (59.6,89.5)	14.1 (12.1,16.5)	42.8 (35.3,51.7)	272.9 (202.6,359.4)	69.1 (50.3,93.6)	167.9 (124.8,222.7)
Total rate of change (%)	1.96	-2.18	1.57	-16.96	-26.95	-18.82	-3.92	-8.52	-4.55
Average annual rate of change (%):									
1990-2005	-0.35	-0.15	-0.27	-0.71	-1.04	-0.77	-0.46	-0.37	-0.41
2005-2019	0.52	0.00	0.40	-0.56	-1.12	-0.65	0.21	-0.24	0.11
**China:**									
1990	207.9 (138.6,297.0)	35.5 (23.3,50.2)	124.7 (82.9,177.7)	52.6 (40.9,82.7)	17.0 (13.7,20.2)	35.5 (29.2,51.3)	260.5 (188.2,352.0)	52.5 (39.8,67.9)	160.1 (117.2,214.7)
2005	171.4 (115.1,243.5)	42.2 (27.2,59.9)	107.8 (71.8,152.6)	95.7 (58.1,109.1)	8.7 (7.7,9.8)	53.0 (34.1,59.9)	267.1 (202.2,340.4)	50.9 (36.0,68.7)	160.8 (122.2,207.7)
2019	201.9 (135.7,288.8)	38.0 (25.0,54.2)	121.1 (81.3,172.4)	74.2 (44.2,93.7)	5.1 (4.0,6.4)	40.0 (25.0,49.9)	276.0 (206.8,365.9)	43.1 (30.0,59.8)	161.1 (119.7,213.9)
Total rate of change (%)	-2.89	6.89	-2.90	40.95	-69.77	12.83	5.97	-17.87	0.59
Average annual rate of change (%):									
1990-2005	-1.28	1.15	-0.96	4.07	-4.37	2.72	0.17	-0.21	0.03
2005-2019	1.18	-0.75	0.83	-1.80	-3.69	-1.99	0.24	-1.18	0.01

YLD – years lived with disability, YLL – years of life lost, DALY – disability-adjusted life years, HICs – high-income countries, UMCs – upper-middle income countries, LMCs – lower-middle income countries, LICs – low-income countries

### YLD-specific

**Figure 1:**
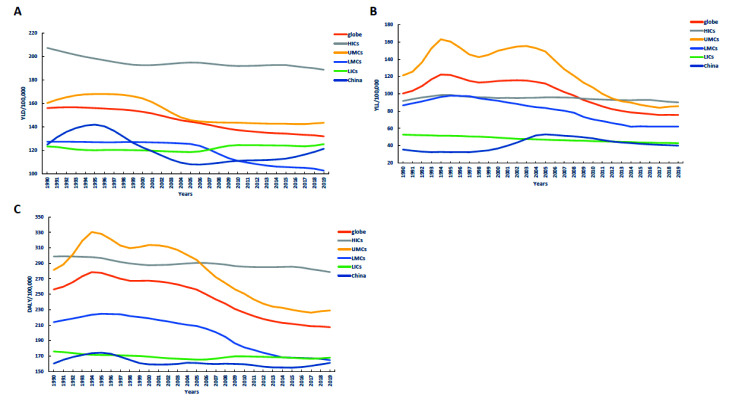
Comparison of the age-standardized YLD, YLL and DALY rate of alcohol use disorders between China and the regions with different income levels over the world from 1990 to 2019. **Panel A.** Comparison of the age-standardized YLD rate caused by alcohol use disorders in China and the regions with different income levels over the world from 1990 to 2019. **Panel B.** Comparison of the age-standardized YLL rate caused by alcohol use disorders in China and the regions with different income levels over the world from 1990 to 2019. **Panel C.** Comparison of the age-standardized DALY rate caused by alcohol use disorders in China and the regions with different income levels over the world from 1990 to 2019.

**Figure 2:**
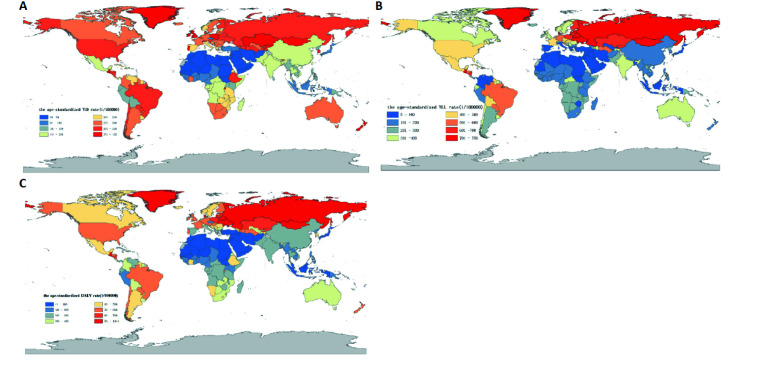
Comparison of the age-standardized YLD, YLL and DALY rate of alcohol use disorders, 1990 to 2019. **Panel A.** Comparison of the age-standardized YLD rate caused by alcohol use disorders,1990 to 2019. **Panel B.** Comparison of the age-standardized YLL rate caused by alcohol use disorders,1990 to 2019. **Panel C.** Comparison of the age-standardized DALY rate caused by alcohol use disorders,1990 to 2019.

The age-standardized YLD rate of AUD showed a four stage trends in the globe, and a six stage trends in HICs, UMCs, LMCs, LICs and China. LMCs experienced the largest changes in the YLD rate of AUD from 1990 to 2019 (average annual percent change (AAPC) = -0.7, 95% confidence interval (CI) = -0.8, -0.7, P < 0.05), with China experienced a higher increase in 1990 to 1993 (annual percent change (APC) = 3.8, 95% CI = 3.2, 4.4, P < 0.05) than other regions, and the rate of decline in China from 1996 to 2002 (APC = -3.4, 95% CI = -3.6, -3.1, P < 0.05) was higher than that in other regions. Through pairwise comparison analysis, the age-standardized YLD rate of AUD in 1990-2019 were statistically significant (*P* < 0.05) between the globe and HICs, globe and UMCs, globe and LMCs, globe and LICs, globe and China, HICs and UMCs, HICs and LMCs, HICs and LICs, HICs and China, UMCs and LMCs, UMCs and LICs, UMCs and China, LMCs and LICs, LMCs and China (**Table 2**, **Figure 1**, panel A and **Figure 2**, panel A).

**Table 2:** Trend analysis of disease burden of alcohol use disorders in China and the regions with different income levels over the world from 1990 to 2019

		Globe			HICs			UMCs			LMCs			LICs			China	
	**Male**	**Female**	**Both**	**Male**	**Female**	**Both**	**Male**	**Female**	**Both**	**Male**	**Female**	**Both**	**Male**	**Female**	**Both**	**Male**	**Female**	**Both**
**YLD**																		
AAPC	-0.6	-0.5	-0.6	-0.5	0.0	-0.3	-0.4	-0.2	-0.4	-0.8	-0.4	-0.7	0.1	-0.1	0.0	-0.1	0.2	-0.1
Lower CI	-0.6	-0.5	-0.6	-0.5	-0.1	-0.3	-0.5	-0.3	-0.4	-0.8	-0.5	-0.8	0.0	-0.1	0.0	-0.3	0.1	-0.3
Upper CI	-0.5	-0.5	-0.5	-0.5	0.0	-0.3	-0.4	-0.2	-0.3	-0.7	-0.3	-0.7	0.1	0.0	0.1	0.0	0.4	0.1
*P*-value	<0.001	<0.001	<0.001	<0.001	0.001	<0.001	<0.001	<0.001	<0.001	<0.001	<0.001	<0.001	0.018	<0.001	0.012	0.101	<0.001	0.230
**YLL**																		
AAPC	-0.8	-1.6	-1.0	-0.3	0.6	-0.1	-0.9	-2.2	-1.1	-0.9	-2.5	-1.1	-0.6	-1.1	-0.7	1.2	-4.0	0.4
Lower CI	-1.0	-1.9	-1.2	-0.4	0.6	-0.2	-1.3	-2.8	-1.6	-1.1	-2.8	-1.3	-0.7	-1.1	-0.8	0.9	-4.4	0.2
Upper CI	-0.6	-1.3	-0.8	-0.2	0.7	0.0	-0.4	-1.6	-0.6	-0.7	-2.3	-1.0	-0.6	-1.0	-0.7	1.6	-3.7	0.7
*P*-value	<0.001	<0.001	<0.001	<0.001	<0.001	0.095	<0.001	<0.001	<0.001	<0.001	<0.001	<0.001	<0.001	<0.001	<0.001	<0.001	<0.001	0.001
**DALY**																		
AAPC	-0.7	-0.8	-0.7	-0.4	0.1	-0.2	-0.6	-0.9	-0.7	-0.8	-1.0	-0.9	-0.1	-0.3	-0.2	0.2	-0.7	0.0
Lower CI	-0.8	-1.0	-0.9	-0.5	0.1	-0.3	-0.8	-1.2	-1.0	-0.9	-1.1	-1.0	-0.2	-0.3	-0.2	0.0	-0.8	-0.1
Upper CI	-0.5	-0.7	-0.6	-0.4	0.1	-0.2	-0.4	-0.7	-0.4	-0.7	-0.9	-0.8	-0.1	-0.3	-0.1	0.4	-0.5	0.2
*P*-value	<0.001	<0.001	<0.001	<0.001	<0.001	<0.001	<0.001	<0.001	<0.001	<0.001	<0.001	<0.001	<0.001	<0.001	<0.001	0.052	<0.001	0.882

YLD – years lived with disability, YLL – years of life lost, DALY – disability-adjusted life years, AAPC – average annual percent change, CI – confidence interval, HICs – high-income countries, UMCs – upper-middle income countries, LMCs – lower-middle income countries, LICs – low-income countries

### YLL-specific

The age-standardized YLL rate of AUD showed a three stage trends in LICs, and a five stage trends in the globe and China, in HICs, UMCs and LMCs showed a six stage trends of change. UMCs experienced the largest changes in the YLL rate of AUD from 1990 to 2019 (AAPC = -1.1, 95% CI = -1.6, -0.6, P < 0.05), with a larger decline in 2004 to 2012(APC = -6.2, 95% CI = -6.7, -5.7, P < 0.05) than other regions, and China experienced a larger increase in the rate of YLL from 1990 to 2004 (APC = 9.2, 95% CI = 8.5, 9.9, P < 0.05) than other regions. Through pairwise comparison analysis, the YLL rate of AUD in 1990-2019 were statistically significant (*P* < 0.05) between the globe and HICs, globe and LICs, globe and China, HICs and UMCs, HICs and LMCs, HICs and LICs, HICs and China, UMCs and China, LMCs and LICs, LMCs and China, LICs and China (**Table 2**, **Figure 1**, panel B, **Figure 2**, panel B).

### YLD /YLL-specific

**Figure 3:**
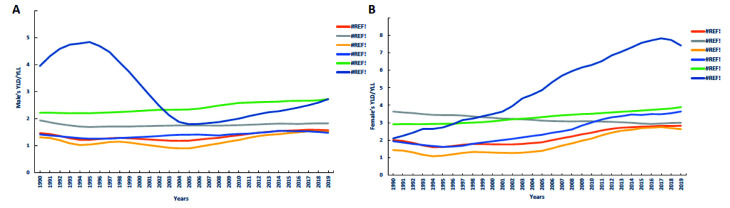
Male and female comparison of the age-standardized YLD rate / YLL rate of alcohol use disorders between China and the regions with different income levels over the world from 1990 to 2019. **Panel A.** Comparison of the age-standardized YLD rate / YLL rate caused by male’s alcohol use disorders in China and the regions with different income levels over the world from 1990 to 2019. **Panel B.** Comparison of the age-standardized YLD rate / YLL rate caused by female’s alcohol use disorders in China and the regions with different income levels over the world from 1990 to 2019.

In 2019, the age-standardized YLD /YLL rate in China (3.0) was higher than those in globe, HICs, UMCs, LMCs, LICs. The age-standardized YLD/YLL rate of male in China showed a downward trend from 1995 to 2005, while showed an upward trend from 2005 to 2019, which was always higher than those in HICs, UMCs, LMCs, and lower than those in LICs. And through pairwise comparison analysis, the male’s YLD/YLL rate of AUD in 1990-2019 were statistically significant (*P* < 0.05) between the globe and HICs, globe and LICs, globe and China, HICs and UMICs, HICs and LMICs, HICs and LICs, HICs and China, UMICs and China, LMICs and LICs, LMICs and China, LICs and China. The age-standardized YLD/YLL rate of female dramatically increased from 1990 to 2017 but slowly decreased from 2017 to 2019. HICs showed different downward trends from 1990 to 2019, while LICs, LMCs, globe and UMCs showed different growth trends. And through pairwise comparison analysis, the female’s YLD/YLL rate of AUD in 1990-2019 were statistically significant (*P* < 0.05) between the globe and HICs, globe and UMICs, globe and LMICs, globe and China, HICs and UMICs, HICs and LMICs, HICs and LICs, HICs and China, UMICs and LICs, UMICs and China, LMICs and LICs, LMICs and China, LICs and China (**Figure 3**).

### DALY-specific

The age-standardized DALY rate of AUD in the globe, HICs, UMCs, LMCs, LICs and China showed a six stage trends of change. LMCs experienced the largest changes in the DALY rate of AUD from 1990 to 2019 (AAPC = -0.9, 95% CI = -1.0, -0.8, P < 0.05), with a larger decline in 2006 to 2010 (APC = -3.3, 95% CI = -3.6, -2.9, P < 0.05) than other regions, and UMCs experienced a larger increase in the rate of DALY from 1990 to 1994 (APC = 4.5, 95% CI = 3.8, 5.1, P < 0.05) than other regions. Through pairwise comparison analysis, the DALY rate of AUD in 1990-2019 were statistically significant (*P* < 0.05) between the globe and HICs, globe and LICs, globe and China, HICs and UMCs, HICs and LMCs, HICs and LICs, HICs and China, UMCs and LICs, UMCs and China, LMCs and LICs, LMCs and China, LICs and China (**Table 2**, **Figure 1**, panel C, and **Figure 2**, panel C).

## DISCUSSION

The rates of YLD, YLL and DALY caused by AUD in China increased in different level, which was harmful to public health and hindered the development of economy and society [30]. In 1990-2019, there were significant difference in the rates of YLL and YLD of different genders in China, and the rates of men were higher than those of women. Two reasons were speculated to be the causes of this phenomenon. The first reason is that men have more unhealthy lifestyle habits and greater life and work stress than women [31-33]. and the second, Chinese traditional culture believes that women’s drinking will reflect their own image and hold negative attitude towards for women drinking behavior [34]. Therefore, preventive interventions targeting men with alcohol use disorders to reduce the burden of alcohol use disorders are more effective than for women [35].

We conducted a comprehensive and systematic analysis of the disease burden of AUD in China and the regions with the different income levels over the word from 1990 to 2019, Our findings underscore the importance of preventing and reducing the burden of AUD in HICs. The age-standardized YLD rate for AUD were higher in HICs than in other regions, which was associated with better access to quality health care and lower mortality rates in HICs. From 1990 to 2019, the AUD age-standardized YLD rates in China and the regions with different income levels over the world were significantly different. The age-standardized YLD rate in globe, China and other income regions showed a decreasing trend with different extents, while LICs has an upward trend (1.57%), which relates to the evidence that rising income inequality accelerates health disparities between countries. In the LICs, the resources available to meet the needs of the people are limited [36], drinkers with AUD do not receive prompt diagnosis and treatment. On the other hand, persistent drinkers are perceived by the public as lacking self-management skills and at risk for social harm [37,38]. When patients were diagnosed with AUD, they felt discriminated and the inferiority reduced their likelihood of seeking treatment [39,40]. The age-standardized YLD rate in China and other income regions decreased to different extents, presumably due to WHO’s effective policies, such as restricting alcohol sales, controlling the drinking environment, early intervention and treatment services [41]. Age-standardized YLD rate (2.90%) declined with the slowest rate in China. This may be related to the fact that in 2005, the China government implemented a relatively loose new tax policy, which canceled the difference on taxes of potato liquor and grain liquor, thus the alcohol consumption of Chinese population increased significantly [42,43], which strongly explains the increasing burden of disease of AUD in China after 2005.

This study shows that compared with different income regions, China's age-standardized YLD/YLL rate (3.02) was higher, implying that YLD is the main contribution to the AUD’s disease burden in China. The government issued two documents of “The Health of China 2030” and “China’s medium and long-term plan for the prevention and treatment of chronic diseases (2017-2025)” and pointed out the suggestions to improve health education on alcohol restriction, to prevent alcohol abuse, to strengthen the monitoring of harmful alcohol use, and to study and improve alcohol taxation policies [44,45]. At present, the control of alcohol use in China is weak and has not formed a comprehensive system of work. The existing alcohol control policy lacks specific goals and measures, so the improvement of the alcohol control policy in China is a long and difficult task [46]. Therefore, China should limit the length of alcohol product advertisement [47], strengthen the propaganda of AUD’s disease, focus on the prevention and treatment of AUD, include the disease in the medical insurance [48-50], and provide free psychological treatment, to prevent its sequelae and complications and improve the quality of life of patients. The results of this study suggest that the disease burden of alcohol use disorders in China is increasing from 2005 to 2019. The government and primary health service organizations should strengthen the study on the risk factors and pathogenesis of AUD, identify the key groups of disease incidence and take intervention measures, especially strengthen the health education for the male high-risk group aged 15-49 and improve their knowledge of three-level prevention of AUD to reduce the risk of diseases.

It is worth noting that there is need to emphasize better guidance in the implementation of public health interventions according to different economic levels to ensure that medical and health care services are used properly for the treatment and management of AUD. In this way, reducing the burden of preventable AUD should be marked as a priority agenda for international and national health care policy makers. Based on the data of GBD 2019, this study analyzed the trend of the burden of disease of AUD between China and the regions with different income levels over the world from 1990 to 2019, the results provide a basis for future policies on AUD. There are some limitations in our study. For example, in GBD 2019’s estimates of YLD, the disability weight is derived from data from several countries around the world, so there is uncertainty in the estimates of diseases in China and the regions with different income over the world [7]. In addition, the comprehensive evaluation of disease burden should include economic, family, social and other factors, and increase the multi-dimensional analysis to improve the accuracy of the results [51].

## CONCLUSION

AUD’s attributable disability burden remains high in the regions with different income levels over the word, which is the main source of the burden of disease. Given the large variations in AUD burden of disease by income level, future strategies to prevent and reduce the burden should be developed and implemented based on country-specific development status. Our findings can serve as a useful reference to inform targeted strategies that account for economic development at both regional and country levels.
